# Ketamine and Lamotrigine Combination in Psychopharmacology: Systematic Review

**DOI:** 10.3390/cells11040645

**Published:** 2022-02-12

**Authors:** Alina Wilkowska, Mariusz S. Wiglusz, Katarzyna Jakuszkowiak-Wojten, Wiesław J. Cubała

**Affiliations:** Department of Psychiatry, Faculty of Medicine, Medical University of Gdańsk, 80-214 Gdańsk, Poland; mwiglusz@gumed.edu.pl (M.S.W.); k.jakuszkowiak@gumed.edu.pl (K.J.-W.); cubala@gumed.edu.pl (W.J.C.)

**Keywords:** ketamine, s-ketamine, r-ketamine, lamotrigine, treatment-resistant bipolar depression

## Abstract

Background and Objectives: Ketamine is a rapid-acting antidepressant with proven efficacy as an add-on agent in unipolar and bipolar treatment-resistant depression. Although many studies have been published, there is still not enough data on the effect of ketamine in combination with other medications. Particularly interesting is the combination of ketamine and lamotrigine, and its potential role in bipolar depression. The aim of this review was to identify animal and human studies in which ketamine and lamotrigine were used together in order to find out if there is scientific ground for combining ketamine and lamotrigine in the treatment of mood disorders. Directions for future studies are presented. Materials and Methods: PubMed and Web of Science were searched. Preferred Reporting Items for Systematic Reviews and Meta-Analyses PRISMA 2020 methodology was applied. Results: Seventeen studies were included for review. Animal studies using models of depression suggested a synergistic effect of ketamine and lamotrigine in combination. Studies on healthy humans showed a reduction in ketamine-induced dissociative symptoms with lamotrigine pretreatment. In a study on patients with depression, ketamine and lamotrigine did not have a stronger antidepressant effect than ketamine alone, but in this study only one ketamine infusion was administered. One case series described the antidepressant and anti-suicidal effect of the combination in two bipolar patients. Available clinical studies on patients with mood disorders did not support the hypothesis that lamotrigine reduces ketamine-induced dissociative symptoms. Conclusions: The results of the analyzed studies were not sufficient to answer any of the stated questions; however, they allowed us to delineate future research directions. The identified animal studies suggested a possible synergistic antidepressant effect of ketamine and lamotrigine. The available clinical studies were not conclusive. No controlled studies on large groups of bipolar patients with multiple ketamine infusions combined with lamotrigine treatment have been published so far. There is some evidence for the reduction of ketamine’s side effects by lamotrigine, and there are reports suggesting that lamotrigine can reduce ketamine craving. More studies with follow-up are needed in order to investigate the ketamine–lamotrigine combination in bipolar patients.

## 1. Introduction

Ketamine is a phencyclidine derivative, acting as an antagonist of the *N*-methyl-d-aspartate receptor. Racemic ketamine consists of R and S enantiomers. It was approved as a rapid-acting general anesthetic in 1970 for intravenous (IV) or intramuscular administration (dose range 1–4.5 mg/kg and 6.5–13 mg/kg, respectively). [1]

Its enantiomer, S-ketamine, an intranasal spray (Spravato), was approved in 2019 by the FDA for add-on treatment of treatment-resistant depression (TRBD), and in 2020 for the treatment of depressive symptoms in adults with major depressive disorder with acute suicidal ideation or behavior [1,2]. Intravenous ketamine is also used off label as an add-on medication for TRD and treatment-resistant bipolar depression, although safety and efficacy of this treatment still requires more studies [1]. It was first administered as a single-dose infusion with the standard of care medication [3,4,5]. Subsequently, repeated ketamine doses were investigated. In one study, multiple ketamine infusions in patients with unipolar and bipolar depression caused about a 50% remission rate [6]. A recent study on ketamine infusions in patients with bipolar depression reported a remission rate of over 60% [7]. In this study, the patients received six infusions and the response and remission rates increased significantly with the number of infusions, suggesting a better effect of repeated dosing. The ketamine dose was 0.5 mg/kg, and it was administered intravenously in 0.9% saline solution for 40 min [7].

The mechanism of antidepressant action in ketamine is still not fully understood, but research suggests it is related to its metabolites. This effect is partly related to the NMDA blockade, but processes concerning other neurotransmitter systems are probably also involved. First, ketamine is converted to R,S norketamine in the liver, then to either R,S dehydronorketamine (DHNK) or (R,S)-hydroxynorketamine (HNK). There are 12 HNKs categorized based on the positioning of a hydroxyl group on the cyclohexyl ring (in position 4, 5, or 6) and their stereochemistry at two stereocenters (R,R,S,S; R,S; or S,R) [8]. Ketamine metabolite (2R,6R)-HNK has appeared to be particularly efficacious in rodent models of depression; it probably does not cause psychotomimetic side effects, and does not have abuse potential. It is probably also capable of binding TrkB and enhancing BDNF signaling, causing the structural potentiation of the synapses [8,9]. Animal studies suggest that factors such as biological sex, circadian rhythm, and prior treatment with other substances such as α 2-adrenoceptor agonists can impact the process of transforming ketamine to HNKs [10].

In another study with more than 200 TRD patients (30 of them with bipolar disorder), the authors found improvement in depressive symptoms and suicidality soon after the fourth infusion [11]. A recent retrospective study on IV ketamine in unipolar and bipolar depression has shown that ketamine has a rapid effect in reducing symptoms such as agitation, irritability, anxiety, and suicidal ideation. These symptoms are very common in depression with mixed features, which is quite common in bipolar disorder and correlates with suicidality [12,13]. To date, two processes involving glutamate transmission appear to be crucial. One is the glutamate ‘surge’ in the prefrontal cortex caused by the inhibition of the NMDA receptor on the GABA interneuron: this effect results in a decreased GABA release and subsequently causes the disinhibition of glutamate release from glutamatergic neurons. The second process is the increase in synaptic density and connectivity which is due to the release of BDNF, and the activation of the mechanistic target of rapamycin complex 1 (mTORC1) [14,15,16]. Studies suggest that ketamine acts also through the α-amino-3-hydroxy-5-methyl-4-isoxazolepropionic acid receptor (AMPAR). It was found that AMPAR antagonists block the synaptic remodeling and the rapid antidepressant effect of ketamine [17]. On the other hand, AMPAR potentiators enhance glutamatergic transmission, produce a rapid antidepressant effect, and increase BDNF production in rodents [18,19]. We need more studies to find out the optimal dosing schedule necessary for sufficient glutamate activation and maintenance of synaptic connectivity changes, as the mechanism is still unclear.

Lamotrigine is an antiepileptic drug from the phenyltriazine class used in the treatment of epilepsy and bipolar disorder. It inhibits voltage-sensitive sodium channels, stabilizes presynaptic neuronal membranes, and inhibits glutamate release [20]. Chronic treatment with lamotrigine enhances extracellular concentrations of serotonin and dopamine in the rat hippocampus [21]. It was also reported that lamotrigine inhibits the synaptosomal uptake of serotonin, noradrenaline and dopamine in rat brain [22].

The proposed mechanisms of action explaining the beneficial effect of lamotrigine in bipolar disorder include inhibiting voltage-sensitive sodium channels, glutamate release and the calcium channel blockade [23]. The antidepressant effect of lamotrigine is probably attributed to the NMDA blockade, which might be involved in this effect.

In an in vitro study, lamotrigine increased the number and activity of AMPA receptors (subunits GluR1 and GluR2) in rat hippocampal neurons. Interestingly, the time- and dose-dependent effect was not observed in the case of valproate [24].

Another effect of chronic lamotrigine administration is the downregulation of arachidonic acid signaling [25]. According to earlier studies, downregulation of the AA cascade increases the expression of BDNF in the brain [26]. Moreover, it seems that the downregulation of the AA cascade increases the expression of AMPA receptors (GluR2, GluR3) [27]. Thus, it can be hypothesized that ketamine-induced AMPA receptor’s upregulation is mediated by the decrease in AA cascade activity.

**Figure 1:**
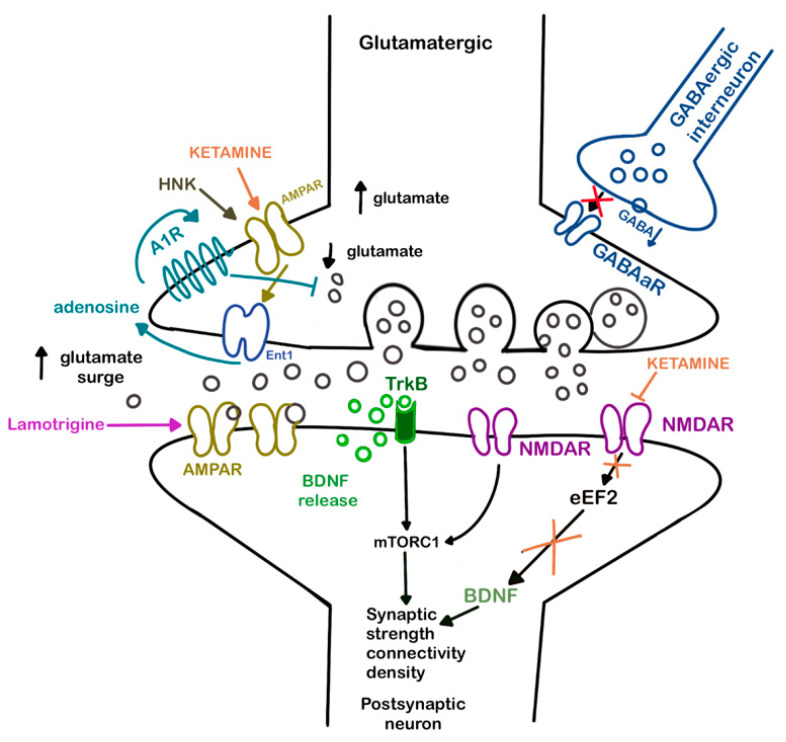
Potential synergistic effect of ketamine and lamotrigine in the treatment of depression.

One study showed that lamotrigine treatment blocked NMDA receptor-initiated arachidonic acid (AA) cascade in rat brain, suggesting its anti-inflammatory effects [28]. There is also evidence from animal studies that chronic administration of lamotrigine can increase brain mRNA and protein levels of BDNF [29,30]. Potential synergistic effect of ketamine-lamotrigine combination are presented in Figure 1.

Bipolar disorder (BD) is an impairing and chronic condition with a prevalence of more than 1% in the general population. Having bipolar disorder significantly increases the risk of suicide. Suicidal acts happen mostly in association with severe depressive or mixed episodes [31,32]. Depression takes a substantial part in patient’s lives [33,34]. The treatment of bipolar depression is often unsuccessful and less effective than treatment of major depressive disorder (MDD) [35]. There is an urgent need for more effective treatment strategies in bipolar depression [36].

According to bipolar disorder treatment guidelines, lamotrigine is one of the first-line agents for bipolar depression both as a monotherapy and an adjunctive treatment, also in resistant cases [37,38]. Lamotrigine is approved by the US Food and Drug Administration (FDA) and European Medicines Agency (EMA) ‘for long-term maintenance treatment of BD type I to prevent relapse in adult patients with predominantly depressive episodes’.

The studies on add-on ketamine treatment in treatment-resistant depression (TRD) and TRBD are mounting, although still little is known about its interactions with medications used in the treatment of bipolar depression. In studies reporting simultaneous use of lamotrigine and ketamine, no serious adverse events were observed, suggesting this combination is rather safe, although further studies with longer follow-up are needed [39].

In this review, we systematically summarize knowledge from mental disorder studies in which ketamine and lamotrigine were used together. This systematic review was written according to the Preferred Reporting Items for Systematic Reviews and Meta-Analyses (PRISMA) statement.

The questions we aimed to address were:

Does lamotrigine increase or decrease the antidepressant effect of ketamine?

Does lamotrigine reduce dissociative symptoms caused by ketamine?

Does lamotrigine decrease the risk of ketamine-use disorder?

## 2. Materials and Methods

The electronic databases MEDLINE and Web of Science were searched on 15 August 2021 for studies which investigated the concomitant use of ketamine and lamotrigine. We used the following entries: ‘ketamine’ AND ‘lamotrigine’, ‘s-ketamine’ AND ‘lamotrigine’, ‘r-ketamine’ AND ‘lamotrigine’, ‘ketamine’ AND ‘lamotrigine’ AND ‘depression’, ‘ketamine’ AND ‘lamotrigine’ AND ‘bipolar depression’. We did not apply any restrictions to date of publishing. Two reviewers—AW and MSW—independently screened the titles, abstracts and full texts. Disparities were discussed between both authors. This review was not registered.

After excluding reviews, animal and human studies in which both medications were used simultaneously were included. Case reports and case series were also analyzed. Three studies were excluded because they were not written in English. We excluded studies on migraine (4), epilepsy (2) and allodynia (1), a letter of response to another article (1), and a hypothesis article (1). A detailed description of the screening process is presented in the flow chart. Information regarding the aim of the study, design, sample size, population characteristics, doses and routes of administration of both medications as well as study outcomes were extracted into 5 tables.

This systematic review was written according to the Preferred Reporting Items for Systematic Reviews and Meta-Analyses (PRISMA) statement.

## 3. Results

**Figure 2:**
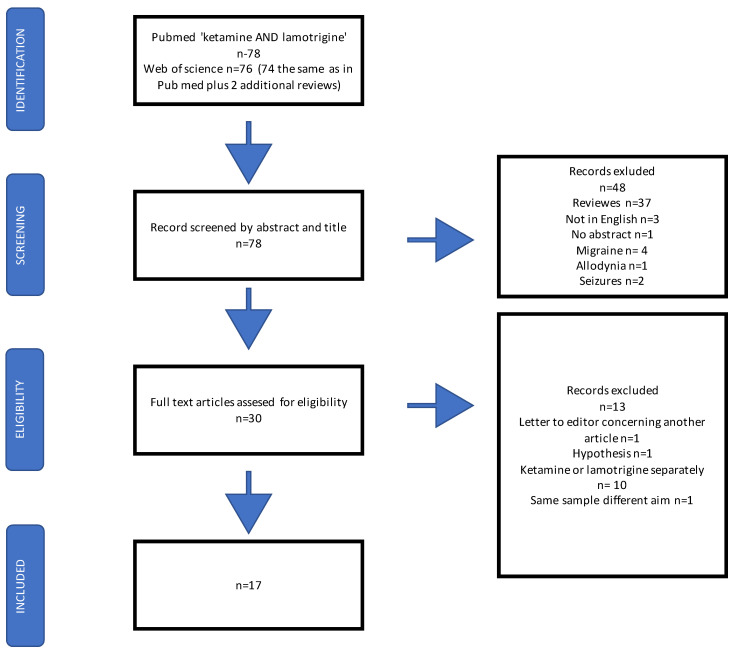
Flow chart representing the search strategy and the process of including studies for analysis.

On 15 August 2021, after applying the above entries, we identified 78 citations in PubMed and Web of Science. The process of screening is presented in detail in the flow chart below (Figure 2).

We included 17 studies: The 14 studies were divided into 4 groups, presented in separate tables: animal studies; human studies on healthy volunteers; human studies on patients with depression; and 1 study on lamotrigine used before ketamine anesthesia (Table 1, Table 2, Table 3 and Table 4). Three of them were case reports or case series and are described in detail in the Supplementary Materials (Table 5).

Seven of these 17 studies were included in a recent systematic review on ketamine interactions with various medications [39].

The raw search results, PRISMA checklist and description of case reports and case series are included in the supplementary materials.

### 3.1. Animal Studies

In the first study, NMDA antagonists (including ketamine) and agonists were used in order to observe their influence on the antidepressant effect of lamotrigine. Co-administration of ketamine (1 mg/kg) and lamotrigine (3 mg/kg) resulted in reduced immobility time in FST (forced swimming test); moreover, the NMDA receptor agonist reversed this effect. The immobile position in the FST reflects helplessness and despair. Reduction of the immobility time is considered an antidepressant-like effect. This observation seems particularly significant in the context of the possible augmentation effect of ketamine and lamotrigine used together in TRBD [40].

The second study investigated the synergistic action of ketamine with lamotrigine, fluoxetine, and quetiapine on behavior, inflammation, and oxidative stress in rats. The combination with lamotrigine caused a lowering of proinflammatory cytokine IL1ß concentration compared to lamotrigine used alone. Lamotrigine and fluoxetine in combination with ketamine reduced lipid damage in the hippocampus compared to ketamine alone. Rats treated with fluoxetine or lamotrigine or these drugs together with ketamine had smaller lipid peroxidation compared with the ketamine group. The authors suggest that lamotrigine and fluoxetine protect mice brain cells from oxidative damage caused by ketamine [41].

A ketamine and lamotrigine combination was also used in studies on prepulse inhibition. Prepulse inhibition (PPI) of a startle response is the reduction in the startle response caused by a low intensity stimulus (the prepulse) which is introduced shortly before the startle stimulus. PPI can be used as a measure of sensorimotor gating in mammals. PPI reflects a process of filtering information and is disturbed in bipolar disorder and schizophrenia. Deficits in PPI can be modelled in rats using a variety of pharmacological agents, including ketamine [42,43]. In a study on mice, the authors found that lamotrigine can increase PPI on its own and prevent ketamine-induced deficit in prepulse inhibition. The authors also suggested that this observation can lead to the development of new anti-manic and mood-stabilizing agents [42]. Contrary results were found in a more recent study, although according to the authors the lack of effect of lamotrigine in this study could be due to strain and species differences [43]. Another study investigated frequency of oscillations in nucleus accumbens (NA). It was reported that ketamine can increase the power of high-frequency oscillations (HFO) in NA [44]. In a study on HFO induced by the intraperitoneal injection of ketamine, animals pretreated with lamotrigine had reduced power and frequency of HFO in nucleus accumbens [56]. The authors suggest that this effect can potentially be connected with the reduction of cognitive and sensorimotor impairments caused by ketamine in rodents and humans [56].

Another line of studies investigated ketamine’s abuse potential and the effect of lamotrigine on ketamine craving. Lee et al. found that lamotrigine (LTG) reduced ketamine craving in rats [45]. Some anti-craving properties of lamotrigine have been observed before in patients with bipolar disorder and cocaine- and alcohol-use disorders [57,58].

### 3.2. Human Studies

#### 3.2.1. Human Studies on Healthy Participants

A healthy volunteer in the following studies was defined as an individual with no known significant health problems. One study on 16 healthy subjects investigated lamotrigine 300 mg administration vs placebo prior to ketamine or placebo. Lamotrigine use correlated with lower CADSS and BPRS scores, and better learning capacity; moreover, lamotrigine increased the mood, elevating effect of ketamine [46]. A randomized double-blind, placebo-controlled study investigating the effect of a single dose of 300 mg lamotrigine administered before ketamine infusion in 19 healthy humans found lower scores in the Clinician-Administered Dissociative States Scale (CADSS) and Brief Psychiatric Rating Scale (BPRS) in the lamotrigine group. Functional magnetic resonance (FMRI) showed significant reduction of the blood oxygenation level-dependent (BOLD) signal in the ventromedial frontal cortex after ketamine administration. This effect correlated with an increase in the CADSS and BPRS scores. Pretreatment with lamotrigine prevented changes in the BOLD signal and decreased CADSS and BPRS scores [47]. In a similar study, Doyle et al. observed the effect of lamotrigine and risperidone on the ketamine-induced BOLD signal in healthy volunteers. They also found an attenuation of ketamine’s effect [48].

Another study (the same sample) on the effect of lamotrigine on resting brain perfusion after ketamine treatment in healthy humans did not support these results. Pretreatment with lamotrigine did not affect ketamine-induced brain perfusion [49].

Another study on 20 healthy males investigated the connectivity pattern of the brain after ketamine treatment preceded with 300 mg of lamotrigine or placebo. This was the same sample described in formerly mentioned studies [48,49]. The authors did not find any alterations of the functional connectivity pattern induced by ketamine in participants receiving lamotrigine before ketamine infusions [50].

#### 3.2.2. Human Studies in Mood Disorders

In a randomized, double-blind study on patients with TRD and healthy subjects, the authors examined the results of 300 mg lamotrigine administered 2 h before ketamine infusion. They used fMRI in order to observe connectivity measure GBC (global signal regression) in various regions of the brain in both groups. They found that pretreatment with lamotrigine correlated with a reduction of ketamine-induced GBCr. Interestingly, lamotrigine did not reduce the scores increased by ketamine in the BPRS and the CADSS [14].

A pilot, randomized, controlled trial on 26 TRD medication-free patients receiving a single dose of 300 mg lamotrigine or placebo prior to single ketamine infusion did not support previous [46] results on lamotrigine attenuating ketamine side effects and no differences in BPRS and CADSS scores were observed. Scores in MADRS did not differ significantly between groups. Ketamine infusion was well tolerated [51]. In this study slow ketamine infusion protocol was used—0.5 mg/kg IV over 40 min. In the former study by Anand [46], higher ketamine dose and a bolus-constant infusion was applied (0.26 mg/kg IV for 1 min followed by 0.65 mg/kg for 90 min); also, there were four ketamine infusions. Another difference was that in this study the participants were TRD patients, not healthy volunteers. Moreover, mood elevation in Anand’s paper was measured by the mood elevation item of YMRS, and not MADRS [46].

A recent case series of 13 patients treated with adjunct ketamine for TRD and TRBD included two subjects who received ketamine infusions and lamotrigine. One case described a patient with bipolar II treatment-resistant depression with suicidality. After 42 infusions of ketamine 0.5 mg/kg over 7 months in combination with lamotrigine 200 mg and aripiprazole 20 mg, the patient’s mood gradually improved and suicidal ideation resolved. It was also possible to take the patient off benzodiazepines. Her cognitive tests also improved; no serious adverse effects were observed. Another TRBD patient with psychotic depression and suicidality received a single ketamine infusion as an add-on to lamotrigine 200 mg, olanzapine 25 mg and bupropion 300 mg. The patient’s active suicidal ideation resolved 24 h after ketamine infusion [53]. 

#### 3.2.3. Human Studies in Substance Use Disorders

Another case report described a 25-year-old patient with ketamine-use disorder using 4–5 g ketamine a day. He was hospitalized twice previously due to this substance abuse, with no effect. During the described hospitalization he was administered lamotrigine tapered slowly to 100 mg a day. Even though the patient continued using ketamine, the craving and the dose of ketamine used decreased radically. The authors suggest that lamotrigine may have a potential in ketamine-use disorder treatment [54].

#### 3.2.4. Human Studies in Anesthesia

A pilot study on 46 adults scheduled for selective surgery each received a single dose of 300 mg of lamotrigine followed by ketamine anesthesia [52]. It was found that the BPRS score was lower in the group receiving lamotrigine (three patients from the placebo group vs one from the lamotrigine group had psychologic disturbances). The results might be difficult to interpret, considering the small groups and the influence of other medications used for anesthesia. One case report described the interplay of ketamine and lamotrigine in a bipolar patient with lamotrigine overdose, who did not respond to ketamine anesthesia after receiving the total dose of 250 mg intravenously over 20 min. The authors also observed euphoria [55].

## 4. Discussion

After analyzing the identified studies on ketamine and lamotrigine combination, we conclude that it is not possible to answer any of the stated questions. Preclinical studies suggest the possibility that lamotrigine could potentiate the effect of ketamine on neuroplasticity and synapse strength, augment the antidepressant effect of ketamine, and reduce lipid damage in the hippocampus [40,41]. The downregulation of AA cascade may increase the activity of AMPA receptors. It can also cause an anti-inflammatory effect by reducing proinflammatory cytokine concentrations [40,41]. It is important to underline that this effect needs time and an effective dose of lamotrigine. Further studies on prepulse inhibition [42,43] are difficult to interpret, but there is evidence for PPI deficit in euthymic patients with bipolar disorder which is modulated through NMDA receptors [59]. Recent research on an isoform of serine/threonine protein kinase (v-AKT) AKT-2 supports this observation. In AKT -2 gene knock out mice, low AKT-2 levels correlated with the impairment of long-term potentiation (LTP) and electrophysiological properties of neurons causing hippocampal circuit disfunction [60]. The expression of AKT-2 is low in patients with bipolar disorder and may be linked to electrophysiological disfunctions observed in patients with this diagnosis [61]. The two included animal studies suggested that lamotrigine can reduce the PPI deficit caused by ketamine, although this subject needs further studies in animal models and clinical research [42,43].

No controlled studies on large groups of patients with bipolar depression treated with both drugs have been performed so far. In such studies, lamotrigine dosing, length of treatment, and the number of ketamine infusions should be considered. Studies on healthy participants suggest that lamotrigine may improve the tolerability of ketamine treatment. There is almost no evidence for an augmented antidepressant effect of ketamine–lamotrigine combination in patients with mood disorders so far. It was observed only in case series and a case report [53,54], but considering the burden of bipolar disorder and the lack of rapid-acting strategies for bipolar depression, there is an urgent need for studies investigating this potentially effective strategy. It is necessary to evaluate the safety of this combination. Some studies used a single administration of 300 mg of lamotrigine, which is disputable due to the risk of serious dermatologic complications such as Stevens–Johnson syndrome [46,47,48]. According to the summary of product characteristics, lamotrigine should be titrated slowly [62]. Another important aspect is the ketamine-abuse potential. A few studies suggest that lamotrigine may reduce this, but this hypothesis certainly needs further investigation [45,54]. This aspect is also significant considering the high comorbidity of bipolar disorder and substance-use disorders [36,46]. The other line of studies suggests the possible reduction of ketamine-induced dissociative symptoms by lamotrigine, but again, such studies have not been performed in the bipolar population [43]. There is a need for further elucidation of the mechanism in which both drugs cause an antidepressant effect, which acquires better animal models for bipolar depression. According to a recent Cochrane review on glutamatergic modulators in bipolar disorder, it is necessary to focus on concomitant medications used with ketamine since mood stabilizers are a crucial part of bipolar disorder treatment, and the available studies are insufficient [63]. Future studies with multiple ketamine infusions and long follow-up times are necessary to investigate the potential synergism of both medications in the treatment of bipolar disorder.

The results of this study should be interpreted with caution. Included studies are based on small groups and due to the lack of data case reports and case series are included. Ketamine in selected studies was used in different models. In a few rodent studies models for psychosis were used; they did not investigate the antidepressant effect of this combination, but we included them because they investigate the potential role of lamotrigine in reducing ketamine’s side effects. The possible synergistic effect of ketamine and lamotrigine has not been addressed in bipolar population so far.

## 5. Conclusions

The results of the selected studies do not allow us to draw firm conclusions. Rodent studies suggest a synergistic antidepressant effect of ketamine and lamotrigine in combination. The available human studies are not conclusive. No controlled studies on large groups of bipolar patients with multiple ketamine infusions combined with lamotrigine treatment have been published so far. There is some evidence for the reduction of ketamine’s side effects by lamotrigine, and there are reports suggesting that lamotrigine can reduce ketamine craving. Randomized controlled studies on larger samples of patients with bipolar depression are necessary to evaluate the antidepressant effect and safety of ketamine–lamotrigine combination in this population.

## Figures and Tables

**Table 1:** Animal studies.

Author	Aim and Study Design	Numer of Subjects	Population	Lamotrigine Dose and Root	Ketamine Dose and Root	Tests and Measures	Outcome
Ostahadi et al. 2016 [40]	To investigate the involvement of NMDA receptors and nitric oxide-cyclic guanosine monophosphate (NO-cGMP) synthesis in possible antidepressant-like effect of lamotrigine in forced swimming test (FST) in mice. NMDA receptor antagonists and agonist were used for exploring the involvement of NMDA receptors in the antidepressant-like effect of lamotrigine. Placebo controlled Mean + SEM	8 in a group	Male Naval Medical Research Institute (NMRI) mice	Lamotrigine 5 mg/kg intraperitoneally	Ketamine (1 mg/kg) intraperitoneally	FST	Co-administration of ketamine (1 mg/kg) and lamotrigine (3 mg/kg) resulted in an antidepressant-like effect in FST, NMDA receptor agonist reversed this antidepressant-like effect.
Reus et al. 2017 [41]	To investigate the synergistic interactions between fluoxetine, quetiapine and lamotrigine in combination with ketamine, Placebo controlled Mean ± SEM	12 in a group, 8 groups	Male Wistar rats	(5.0 mg/kg) intraperitoneally	(5.0 mg/kg) intraperitoneally	FST OFT ST	The levels of IL-1ß were reduced in the serum of rats receiving lamotrigine in combination with ketamine, compared to lamotrigine group No difference was observed in behavioral tests results Rats treated with fluoxetine and lamotrigine or with the combination of ketamine with fluoxetine or lamotrigine had a reduction in the lipid peroxidation, compared with group that received only ketamine.
Brody 2003 [42]	To assess the ability of lamotrigine to reduce the PPI– disruptive effects of ketamine and the dopaminergic agent amphetamine in two inbred mouse strains Placebo controlled Mean ± SEM.	Not stated	two inbred mouse strains, C57BL/6J and 129SvPasIco.	Lamotrigine (0,6.7, 13, or 27 mg/kg) or a combination of lamotrigine (27 mg/ kg) and either d-amphetamine or ketamine	ketamine (100 mg/kg)	PPI	In the 129SvPasIco mice, lamotrigine reversed the ketamine-induced PPI deficit, without altering PPI in control mice. In C57BL/6J mice 27 mg/kg lamotrigine generally increased PPI in both control and ketamine-treated mice.
Cilia 2007 [43]	To investigate the effects of antipsychotics and lamotrigine upon ketamine-induced PPI deficits in rats. Placebo controlled Cross over design Mean ± SEM % PPI	12 in a group	Male Sprague Dawley rats	lamotrigine (3–30 mg/kg p.o.; 60 min ptt)	Ketamine (1–10 mg/kg s.c; 15 min ptt)	PPI	Ketamine significantly increased startle amplitude at all doses tested. Lamotrigine failed to significantly attenuate ketamine-induced PPI Deficits. It may be possible that the lack of effect of lamotrigine (3–10 mg/kg) in this study was due to strain and species differences.
Hunt et al. 2008 [44]	To examine if lamotrigine would disrupt ketamine-enhanced HFO Rats were pretreated with either saline or lamotrigine followed by injection of ketamine. A separate group received a unilateral intra-NAc infusion of lamotrigine followed by systemic injection of ketamine Placebo controlled Mean ± SEM	32	Wistar rats	Lamotrigine 0.1 mL/100 g rat weight intraperitoneal injection 3 doses 2 mg/kg, 6.7 mg/kg 20.1 mg/kg	intraperitoneal injection of 25 mg/kg ketamine	HFO	Lamotrigine pretreatment had a significant effect on ketamine-induced behavioral activation Systemic injection of a high dose of lamotrigine (20.1 mg/kg) reduced the power and frequency of ketamine-enhanced HFO. Local infusion of lamotrigine into the NAc did not significantly affect ketamine-induced HFO.
Lee 2019 [45]	To find out if lamotrigine can reduce the motivation for ketamine use and ketamine seeking behavior in rats. Intravenous ketamine self-administration paradigm was used. Placebo controlled Mean ± SEM	Not stated	Male Sprague-Dawley rats	lamotrigine orally 10 mg/kg 30 mg/kg	intravenous ketamine (0.5 mg/kg/infusion)	IV ketamine self-administration paradigm	Lamotrigine 30 mg/kg attenuated the reinforcing efficacy of ketamine and educed ketamine craving and relapse risk

IL-1ß = interleukine 1ß; NO-cGMP = Nitric oxide-cyclic guanosine monophosphate; PPI = prepulse inhibition; HFO = high frequency oscillations; FST;forced swimming test; OFT = open field test, ST = splash test; NMDA = *N*-Methyl-d-aspartate; SEM = standard error of the mean; SD = standard deviation.

**Table 2:** Human studies on healthy participants.

Author	Aim and Study Design	Number of Participants	Population	Lamotrigine Dose and Root	Ketamine Dose and Root	Tests and Measures	Outcome
Anand et al. 2000 [46]	To test if lamotrigine can reduce neuropsychiatric effects of ketamine Randomized, double blind Mean ± SEM	19, 16 completed the study	Healthy humans	Lamotrigine 300 mg single dose 2 h prior to ketamine	0.26 mg/kg iv in 1 min followed by 0.65 mg/kg for 90 min Four infusions	YMRS HVLT CADSS BPRS	Lamotrigine caused further increase in ketamine-induced mood elevation (YMRS) and decrease in ketamine-induced impairment of learning (HVLT) and dissociative symptoms (CADSS). Significant decrease in ketamine-induced positive and negative symptoms (BPRS) was observed.
Deakin et al. 2008 [47]	To determine the role of increased glutamate release as an effect of ketamine with the use of lamotrigine. Randomized, double blind, placebo controlled, crossover, counter balanced-order trial SD	21, 19 completed the study	Healthy right-handed humans	Lamotrigine, 300 mg, oral, 2 h prior to ketamine	0.26 mg/kg IV in 1 min followed by 0.25 mg/kg/h Single infusion	CADSS BPRS BOLD	Lamotrigine pretreatment resulted in significantly lower BPRS and CADSS scores. Several areas showing BOLD signal responses to ketamine in the ketamine-placebo experiment also showed significantly greater response to ketamine after placebo infusion compared to lamotrigine infusion.
Doyle et al. 2013 [48]	To test the hypothesis if lamotrigine or risperidone can reduce ketamine-induced glutamate release. Randomized, double blind, placebo controlled, crossover trial Least Square Mean (95%CI) Difference (95% CI)	20, 16 completed the study	Healthy humans	Lamotrigine 300 mg oral, or placebo, 4.75 h prior to ketamine	Ketamine 0.12 (mean) mg/kg iv during 1 min followed by 0.31 mg/kg/h Four test days 1control and 3 ketamine infusions, two of which included pretreatment with lamotrigine or risperidone	BOLD	A significant positive and negative BOLD response was revealed to ketamine infusion. For the positively responding regions, pretreatment with lamotrigine resulted in attenuation of the ketamine responses. For the negatively responding regions the attenuating effect of lamotrigine was weak.
Shcherbinin et al. 2015 [49]	To assess the effects of ketamine, risperidone and lamotrigine, on resting brain perfusion Randomized, double blind, placebo controlled, crossover trial Accuracy (%)	20, 16 completed the study Same sample as Doyle et al. (2013) and Joules et al. (2015)	Healthy humans	Lamotrigine 300 mg oral, or placebo, prior to ketamine	Ketamine 0.12 mg/kg iv during 1 min followed by 0.31 mg/kg/h Four test days	Resting brain perfusion	Lamotrigine had no significant effect on resting brain perfusion.
Joules et al. 2015 [50]	To investigate the functional connectivity effects of ketamine with pharmacological magnetic resonance imaging (phMRI) and the potential modulation of these effects by pre-treatment with lamotrigine and risperidone Randomized, double blind, placebo controlled, crossover trial Accuracy (%)	20, 16 completed the study Same sample as [48] and [49].	Healthy humans	Lamotrigine 300 mg oral, or placebo, 4.75 h prior to ketamine	Ketamine 0.12 (mean) mg/kg IV in 1 min followed by approximately 0.31 mg/kg/hb.c Four test days	Functional connectivity	No evidence of a significant modulation effect of the ketamine-induced degree-centrality pattern by lamotrigine

BOLD blood oxygenation level- dependent; BPRS = Brief Psychiatric Rating Scale; CADSS = Clinician-Administered Dissociative States Scale; GBCr = global brain connectivity with global signal regression; HVLT = Hopkins Verbal Learning Test; IDS-C30 = Inventory of Depressive Symptomatology—Clinician Rated; IV = intravenous; MADRS = Montgomery-Asberg Depression Rating Scale; MDD = major depressive disorder; NMDA = *N*-methyl-d-aspartate; TRD = therapy resistant depression; vPFC = ventral prefrontal cortex; YMRS = Young Mania Rating Scale.

**Table 3:** Human studies in mood disorders.

Author	Aim and Study Design	Numer of Subjects	Population	Lamotrigine Dose and Root	Ketamine Dose and Root	Tests and Measures	Outcome
Abdallah et al. 2017 [14]	To investigate prefrontal GBCr in treatment-resistant depression (TRD) at baseline and following treatment. Randomized, double blind, placebo controlled crossover trial Mean ± SEM	22 patients with TRD 29 healthy control	Patients with TRD, healthy controls	Lamotrigine 300 mg oral, or placebo, about 2 h prior to ketamine	Ketamine 0.23 mg/kg IV in 2 min followed by 0.58 mg/kg for approximately 70 min Single infusion	BPRS CADSS GBC	Ketamine significantly increased BPRS and CADSS scores but pretreatment with lamotrigine had no significant effect on the ketamine-induced increases. Lamotrigine significantly reduced the ketamine-induced GBCr surge by inhibition of glutamatergic transmission. Ketamine did not significantly reduce vPFC GBCr in TRD subjects but it did reduce vPFC GBCr in healthy subjects. Following pretreatment with lamotrigine, ketamine showed no significant effects on the GBCr in the vPFC.
Mathew et al. 2010 [51]	To replicate the acute efficacy of single-dose intravenous (i.v.) ketamine; test the efficacy of the glutamate-modulating agent riluzole in preventing postketamine relapse; and examine whether pretreatment with lamotrigine would attenuate ketamine’s psychotomimetic effects and enhance its antidepressant activity Randomized, double blind, placebo controlled trial Response and remission rates (%) Mean ± SD	26	Medication free patients with a diagnosis of MDD, of at least moderate severity and nsufficient response to >2 adequate antidepressant trials in the current episode.	Lamotrigine 300 mg oral, or placebo, 2 h prior to ketamine infusion	Ketamine 0.5 mg/kg iv for 40 min Single infusion	BPRS CADSS MADRS	Lamotrigine pretreatment did not attenuate side-effects associated with ketamine. There was no difference detected in MADRS scores and no differences on BPRS positive symptoms between lamotrgine and placebo treatment groups. No difference in CADSS scores was found.

TRD = treatment resistant depression; TRBD = treatment resistant bipolar depression; MADRS = Montgomery-Asberg Depression Rating Scale,;BPRS = Brief Psychiatric Rating Scale; CADSS = Clinician-Administered Dissociative States Scale; GBCr = global brain connectivity with global signal regression; vPFC = ventral prefrontal cortex; SEM = standard error of the mean; SD = standard deviation.

**Table 4:** Human study in substance use disorder.

	Aim and Study Design	Number of Participants	Population	Lamotrigine Dose and Root	Ketamine Dose and Root		Outcome
Maheshwari et al. 2021 [52]	To test the hypothesis that a single dose of lamotrigine 300-mg given before surgery reduces ketamine-induced psychological disturbances. Pilot randomized double-blind trial Relative risk (95% CI)	46 adults (23 Lamotrigine, 23 placebo)	Patients scheduled for elective noncardiac surgery with general anesthesia	Lamotrigine 300 mg/d Single dose	ketamine 1 mg/kg bolus at induction of anesthesia followed by a 5-μg/kg/min infusion which was stopped at the end of surgery.	BPRS	No patients randomized to lamotrigine had psychologic disturbances measured with BPRS, whereas 3 (14%) assigned to placebo did.

TRD = treatment resistant depression; TRBD = treatment resistant bipolar depression; 16 item Self-Report Quick Inventory of Depressive Symptomatology (QIDS-SR16); Beck Depression Inventory (BDI).

**Table 5:** Human studies in anesthesia.

Author	Aim and Study Design	Numer of Subjects	Population	Lamotrigine Dose and Root	Ketamine Dose and Root	Tests and Measures	Outcomes
Chan et al. 2018 [53]	Case series	13 TRD and TRBD patients, 2 of them (TRBD) received ketamine and lamotrigine		Patient 1 Lamotrigine 200 mg/d oraly Patient 2 Lamotrigine 200 mg/d oraly	Patient 1 Ketamine 0.5 mg/kg iv 42 infusions over 7 months Patient 2 Ketamine 0.5 mg/kg iv Single infusion	QIDS-SR16 BDI	Patient 1 Mood, suicidality and cognitive functions improved Patient 2 Active suicidal ideation resolved 24 h after ketamine infusion
Huang et al. 2016 [54]	Case report	1	25 years old male with ketamine use disorder	Lamotrigine 100 mg/d orally (slow titration)	He used ketamine 6–10 times almost daily (total, 4–5 g/day) by smokind and snorting	Not stated	one case of ketamine use disorder who experienced a great reduction in craving and ketamine use after lamotrigine treatment
Kornhal and Nielsen 2014 [55]	Case report describing Failure of Ketamine Anesthesia in a patient with lamotrigine overdose	1 bipolar patient	-	Lamotrigine intoxication, serum concentration was 191.9 micromol/Ltherapeutic reference area is 10–60 micromol/L.	total ketamine dose of 250 mg iv	Not stated	Despite being injected with a total of 250 mg ketamine, The patient presented no signs of dissociative anaesthesia.

TRD = treatment resistant depression; TRBD = treatment resistant bipolar depression; 16 item Self-Report Quick Inventory of Depressive Symptomatology (QIDS-SR16); Beck Depression Inventory (BDI).

## Data Availability

The search strategy is presented in methodology section and on the flow chart.

## Supplementary Materials

The following supporting information can be downloaded at: https://www.mdpi.com/article/10.3390/cells11040645/s1, Prisma checklist, raw pubmed results [64].
